# Implementation of an enhanced recovery after surgery (ERAS) program in patients undergoing cytoreductive surgery and hyperthermic intraperitoneal chemotherapy: study protocol for a prospective multicenter interventional trial (EPICH study)

**DOI:** 10.1515/pp-2024-0033

**Published:** 2025-04-25

**Authors:** Manuela Robella, Eva Pagano, Lisa Giacometti, Armando Cinquegrana, Luca Pellegrino, Andrea Evangelista, Alessandra Saliva, Alessandro Cerutti, Felice Borghi

**Affiliations:** Unit of Surgical Oncology, Candiolo Cancer Institute, FPO - IRCCS, Candiolo (TO), Italy; Unit of Cancer Epidemiology, University of Torino, CERMS and CPO-Piemonte, Turin, Italy; Unit of Clinical Epidemiology, Azienda Ospedaliero Universitaria Città della Salute e della Scienza di Torino, Turin, Italy; Clinical Epidemiology Unit, AOU Città della Salute e della Scienza di Torino e CPO Piemonte, Turin, Italy; Anesthesia and Intensive Care Unit, Candiolo Cancer Institute, FPO – IRCCS, Candiolo (TO), Italy

**Keywords:** ERAS, HIPEC, enhanced recovery, cytoreductive surgery, peritoneal surface malignancies, intraperitoneal chemotherapy

## Abstract

**Objectives:**

This study aims to evaluate the clinical impact of introducing an Enhanced Recovery After Surgery (ERAS) protocol in the management of patients undergoing cytoreductive surgery (CRS), with or without hyperthermic intraperitoneal chemotherapy (HIPEC). By addressing a population at high risk of postoperative complications and delayed recovery, the study seeks to determine whether ERAS can improve short-term outcomes, optimize perioperative care, and promote faster and safer recovery in a standardized, evidence-based manner across multiple centers.

**Methods:**

The EPICH study is a multicenter, prospective, interventional trial conducted across 20 centers in Italy. A total of 300 patients undergoing CRS±HIPEC will be enrolled in two sequential phases: standard perioperative care followed by ERAS protocol implementation. The primary endpoint is the mean hospital length of stay (LOS). Secondary endpoints include postoperative complications, ICU admission, readmission rates, bowel function recovery, mortality, and patient-reported quality of recovery. The ERAS protocol includes prehabilitation, anemia and nutritional optimization, intraoperative fluid and pain management, and early mobilization and oral feeding. Data will be analyzed using random-effects linear models to account for center-level variation and confounding factors.

**Results:**

Preliminary evidence suggests that the ERAS protocol may help reduce mean hospital LOS, postoperative complications, and ICU stays, as well as support faster bowel recovery and improved patient-reported outcomes—findings that this study seeks to validate.

**Conclusions:**

The EPICH study could provide robust evidence supporting the adoption of ERAS as the standard of care for patients undergoing CRS±HIPEC, with potential benefits in terms of improved recovery, reduced complications, and decreased healthcare resource utilization.

## Background

The E.R.A.S (Enhanced Recovery After Surgery) program was developed with the objective of optimizing the care pathway of patients who are candidates for elective major surgery, with the aim of facilitating an earlier post-surgery recovery. The protocol is comprised of a set of strategies and procedures based on scientific evidence from various disciplines beyond surgery, including anesthesia, nutritional care and rehabilitation. The various specialist must interface and coordinate with each other in order to minimize perioperative stress and promote a rapid recovery of physiological functions in the postoperative period. It has been demonstrated that this multimodal approach, which originated in colorectal surgery in the second half of the 1990s, has reduced morbidity rate, convalescence and length of stay (LOS) in standard colorectal operations [1], [2], [3], [4]. Furthermore, it has been adopted by other surgical disciplines, such as gynecology and urology [5], [6], [7].

Cytoreductive surgery in combination with hyperthermic intraperitoneal chemotherapy (HIPEC) has now been established as a valid therapeutic option for patients with peritoneal surface malignancies. It represents the gold standard for the treatment of pseudomyxoma peritonei and peritoneal mesothelioma and may also be a potential strategy for selected patients with peritoneal metastasis of ovarian or gastrointestinal origin. The objective of surgical cytoreduction is the removal of macroscopically visible disease (or the reduction of it to leave minimal residual disease). The combination with HIPEC allows for the removal of any microscopic residual disease that may still be present. Despite the well-established benefits of this combined treatment, the literature reports non-negligible postoperative morbidity and mortality rates due to the extent of the surgical procedures and the addition of intraperitoneal chemotherapy, which impedes the healing process and recovery of organ function. A direct consequence of these effects is a slowed recovery of intestinal motility and a prolonged length of hospitalization, which results in higher costs.

The ERAS Society has formalized guidelines for enhanced recovery after surgery for a variety of surgical procedures [8], [9], [10], [11]. Additionally, guidelines for patients with peritoneal surface malignancies undergoing cytoreductive surgery with or without HIPEC have recently been published [12], 13]. A substantial body of evidence demonstrates that the implementation of the ERAS program in this surgical context has resulted in a reduction in postoperative complications and hospitalization times, while concurrently enhancing patient satisfaction [14], [15], [16], [17]. In light of these outcomes, our Institute, where the ERAS program is already routinely employed for elective digestive [18] and gynaecological surgery [19], has expanded this protocol to encompass the perioperative management of patients with peritoneal malignancies.

A survey was conducted at the end of 2023 at 21 Italian referral centers specialized in the treatment of peritoneal surface malignancies. The results demonstrated a high level of interest in the systematic application of the ERAS program in this field (76.2 %). However, various difficulties in the application of the guidelines were also identified, including the high number of items, organizational problems and the low level of evidence in terms of efficacy [20]. In order to develop a suitable protocol, a second survey was conducted with the objective of reviewing all the items proposed in the guidelines and of selecting those that registered a high level of consensus regarding their effectiveness and applicability in this context. A shared ERAS protocol has been identified. The EPICH study is a multicentric interventional pre-post study aimed to evaluate the feasibility of the ERAS approach implementation for patients who are candidates for cytoreductive surgery and HIPEC and to assess its impact on the patients’ outcomes.

## Patients and methods

### Study design

EPICH study is a multicenter, prospective, interventional, cohort study with a pre-post design assessing the perioperative management according to the ERAS protocol in patients with peritoneal metastasis undergoing cytoreductive surgery with or without HIPEC.

Approximately 300 patients will be enrolled in 20 centers over 12 months. In the first period (approximately 4 months, 100 patients planned) the usual perioperative procedures will be described. In the second phase (approximately 8 months, 200 patients expected), the participating centers will apply the defined and agreed ERAS protocol.

The following elements of the ERAS protocol will be monitored during the study: prehabilitation and preoperative counselling, dietary assessment and possible nutritional support, antibiotic prophylaxis and bowel preparation, preoperative fasting management and postoperative resumption of fluid/solid intake per os, management of infusions and intra/postoperative analgesic therapy.

### Population and selection criteria

The EPICH study will recruit patients with peritoneal surface malignancies candidates for cytoreduction surgery, whether or not associated with HIPEC. Eligibility criteria are reported in Table 1.

**Table 1:** Eligibility criteria for EPICH Study.

Inclusion criteria	Exclusion criteria
– Written informed consent signed prior to procedure – Diagnostic histological/cytological examination of advanced solid tumour with documented peritoneal disease from the following neoplasms: – Tumour of gynecological origin – Gastric cancer – Tumour of intestinal origin – Age between 18 and 75 years – Performance status according to ECOG≤1 – ASA score ≤3	– Lack of signed written informed consent – ASA score >4 – Palliative or other unplanned surgery – Severe renal/liver/cardiac failure; a recent myocardial infarction, or severe arrhythmia are excluded from the study. – Immunosuppression, whether due to underlying disease or immunosuppressive therapy, or primary immunodeficiency.

## Intervention

### Establishing an ERAS team

In order to comply with the standards set forth by the ERAS program, a team comprising surgeon, anesthetist, dietician and nurse (the physiotherapist intervenes at the request of the medical staff if necessary) must be assembled. This team is responsible for implementing the protocols and principles set forth in the program, with the aim of facilitating early recovery. A structured implementation phase, including training and adaptation of workflows, will precede the ERAS intervention. This will ensure a standardized and effective transition across participating centers.

**Figure 1:**
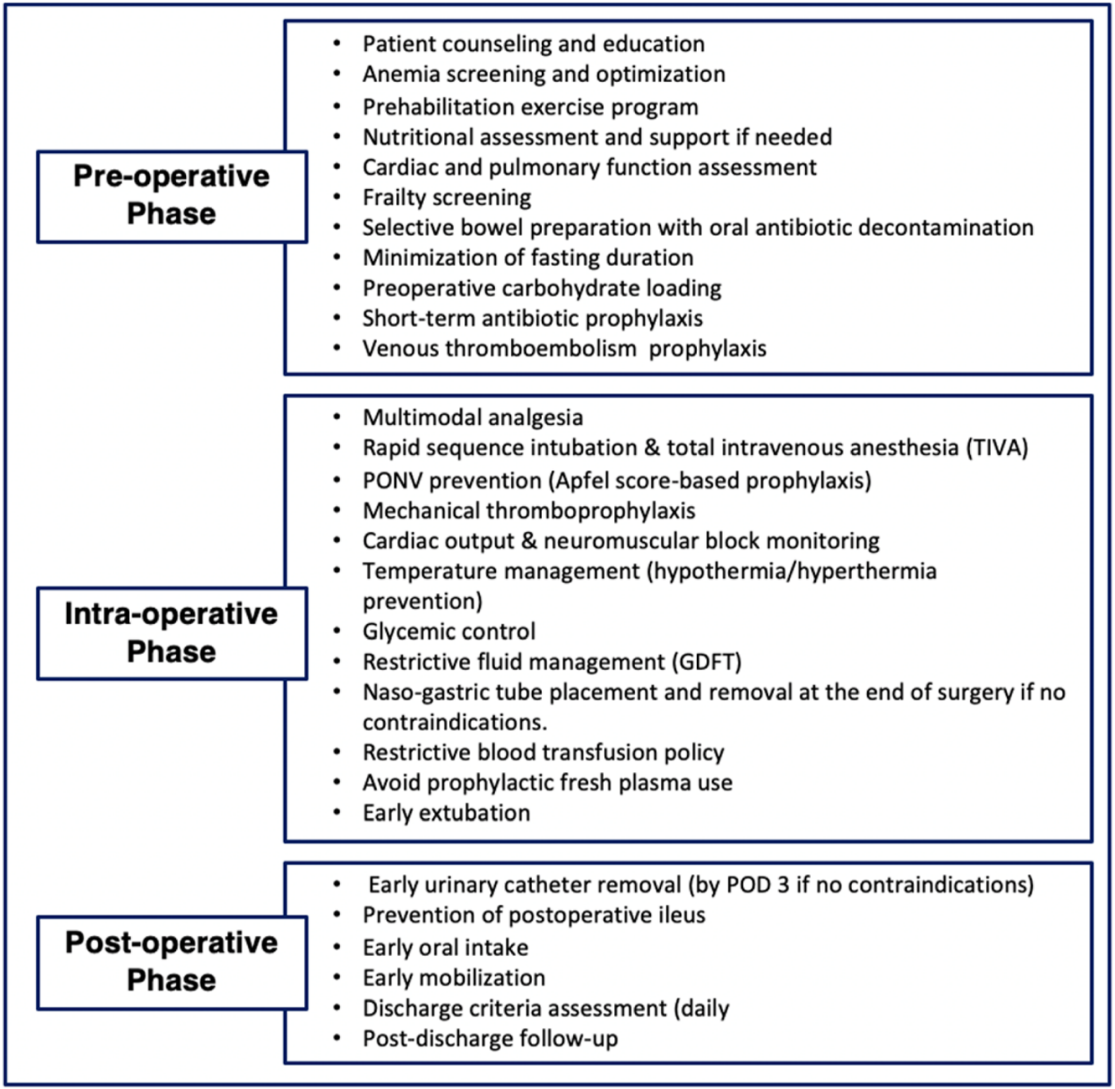
Structured ERAS pathway for patients undergoing CRS ± HIPEC: pre-, intra-, and postoperative phases.

The protocol precisely follows each recommendation of the official guidelines published by the ERAS Society for the perioperative management of patients undergoing cytoreductive surgery with or without HIPEC. Figure 1 illustrates the flowchart of pre-, intra-, and postoperative items.

### Pre- operative phase management

– Pre-operative counselling (to be conducted by the team during a designated meeting): the patient is informed of the details of the ERAS program which encompasses corrective interventions aimed at cessation of smoking and alcohol abuse.
– Screening for anemia: it should be conducted at least 4 weeks prior to CRS ± HIPEC. In the event that anemia is identified, prompt medical therapy with oral or intravenous iron, folate/vitamin B12 implementation or treatment with erythropoietin should be recommended. Preoperative transfusions should be considered in cases of severe refractory situations.
– A prehabilitative exercise program, preferably integrated with other interventions (nutritional or anxiety control), should be initiated.
– Assessment of nutritional status based on the Malnutritional Universal Screening Tool (MUST). In malnourished patients or patients at risk of malnutrition, nutritional and protein support (oral>enteral>parenteral) should be routinely indicated for at least 5 days, and in the case of severe malnutrition, up to 14 days. In all patients, pre-operative immunonutrition administration for 5–7 days before surgery may be indicated.
– Assessment of cardiac function, comprising a cardiological examination and echocardiography.
– Comprehensive respiratory assessment, comprising a pulmonary examination and spirometry with DLCo and RV assessment (especially if diaphragmatic peritonectomy is planned); additionally, a screening for obstructive sleep apnea syndrome (OSAS) could be considered.
– Screening process for frailty. Frailty is defined as a state of increased vulnerability and impaired return to homeostasis after physiological stress, and is correlated with worse outcomes after major surgery. The Clinical Frailty Scale (CFS) is a comprehensive assessment tool comprising nine levels that classify a patient’s clinical condition from “fit” to “terminally ill”. The CFS has been demonstrated to be an independent predictor of readmission, disability, and mortality. In the event that a patient presents with a frailty score>4, and this score is not amenable to modification, it may be inadvisable to pursue CRS ± HIPEC.
– It is recommended that long-acting sedatives and anxiolytics should be avoided prior to CRS ± HIPEC.
– Bowel preparation should be performed in conjunction with oral antibiotic decontamination (Neomycin 2 g and Metronidazole 2 g, or similar) exclusively in instances of multiple bowel resections or sigmoidectomy/rectal anterior resection. An enema procedure should be conducted in the evening before surgery. In the event of a sigmoid or rectal resection, perioperative rectal clyster with betadine solution is recommended.
– Patients are permitted to consume only clear liquids up to 2 h prior to surgery, while solid foods must be avoided for a minimum of 6 h.
– The administration of carbohydrates up to 2 h prior to the induction of anaesthesia has been demonstrated to reduce postoperative insulin resistance and perioperative discomfort, including anxiety.
– The administration of antibiotic prophylaxis is contingent upon the guidelines set forth by the respective institution, provided that the regimen is either short-term (within 24 h of surgery) or a single dose (which may be repeated during the surgical procedure).
– Antithrombotic prophylaxis: low-molecular-weight heparin should be administered the evening preceding the surgical procedure.
– It is recommended that the administration of bevacizumab or other anti-angiogenic drugs be suspended for a minimum of 5 weeks prior to CRS ± HIPEC.

### Intra-operative phase management

– The administration of preoperative multimodal analgesia, comprising paracetamol 1 g and parecoxib 40 mg, is recommended.
– Rapid sequence intubation is recommended.
– The use of total intravenous anesthesia (TIVA) as an alternative to inhalation anesthesia is recommended as a means of preventing postoperative nausea and emesis.
– The prevention of postoperative nausea and vomiting (PONV) in the presence of a Apfel score of ≥2 s is of paramount importance. An Apfel score 2 indicates that prophylaxis is at the discretion of the surgical team. For an Apfel score ≥3, the recommended treatment is dexamethasone 4–8 mg at the time of induction and ondansetron 4 mg at the time of awakening. Post-operatively, ondansetron 8 mg should be administered every 8 h for the first 24 h.
– Placement of a peridural catheter (T5-T11, low doses of local anesthetic and opioids) is recommended to be maintained for at least 72 h after CRS ± HIPEC in order to achieve better analgesic control by minimizing the need for intravenous opioid medication. A regional blockade may reduce the stress response by reducing insulin resistance. It may be beneficial to consider the potential use of one or more non-opioid agents, such as dexmedetomidine, magnesium sulphate, lidocaine and ketamine, as a form of supplementation.
– It is recommended that protective mechanical ventilation with low tidal volumes be employed.
– Urinary catheter positioning.
– Naso-gastric tube (NGT) placement and removal at the end of surgery in the absence of risk factors for delayed gastric emptying.
– Mechanical thromboprophylaxis (intermittent pneumatic compression) is recommended.
– Monitoring of cardiac output through advanced systems, such as EmoSphere and PulsioFlex Monitor.
– Monitoring of neuromuscular block.
– Prevention of intraoperative hypothermia (defined as a temperature below 36 °C) through active heating devices, a room temperature of 21 °C and/or a closed circuit (fresh gas <1 lt/min), infusions and flushing fluids.
– Prevention of intraoperative hyperthermia (defined as a temperature exceeding 41 °C) through active measures, including the use of forced ventilators, cold packs, and environmental settings.
– Intraoperative glycemic control to be maintained within the range of 140–180 mg/mL.
– Intraoperative restrictive hydration protocol as follows: infusion rate of 5 mL/kg/h should be maintained, with a crystalloid-to-colloid ratio of 1:1 or 2:1. The use of Goal Directed Fluid Therapy (GDFT) or a zero-water balance, based on the patient’s risk profile, is recommended in order to maintain adequate urinary output (1 mL/kg/h). During the perfusion phase, it is essential to increase fluid administration in order to maintain a minimum diuresis of approximately 500 mL/60 min. The administration of vasoconstrictors is advised for patients exhibiting normovolaemic hypotension.
– Restrictive policy in terms of blood transfusions, with a threshold hemoglobin level of 8 g/dL.
– It is recommended that fresh plasma be avoided in prophylactic use.
– Early extubation in the absence of contraindication.

### Post-operative phase management

– In the absence of contraindications, the removal of the urinary catheter should be undertaken as soon as possible, ideally by the morning of the third postoperative day (POD).
– The use of laxatives, prokinetics and additional measures (such as coffee and chewing gum) alone or in combination is recommended as a means of preventing postoperative paralytic ileus.
– Following the removal of the peridural catheter, a combined analgesic regimen should be initiated, comprising paracetamol, non-steroidal anti-inflammatory drugs and, if necessary, minor opiates.
– It is recommended that patients consume clear fluids on the day of surgery, provided there are no contraindications.
– A light solid diet may be commenced from the first (POD), provided there are no contraindications. It may be beneficial to consider the addition of oral supplements alongside the usual diet during the initial five PODs.
– Food diary to be completed on a daily basis.
– Parenteral nutrition with high-protein bags is to be administered for a minimum of 3 days, with a maximum of 7 days permitted. The total volume of infusions should be between 1 and 2 mL/kg/h, with a gradual reduction based on the patient’s oral intake and calories ingested, as assessed by the dietitian.
– Postoperative blood glucose monitoring is to be conducted with the objective of maintaining levels between 140 and 180 mg/dL.
– Pharmacological thromboprophylaxis with LMWH is to be maintained for up to 4 weeks following surgery.
– Early mobilization is to be initiated on POD 2 with the aim of achieving 2 h of out-of-bed activity, and 6 h on the third POD.
– The measurement of the criteria for determining whether a patient is fit for discharge should be conducted on a daily basis. The following criteria must be met for a patient to be deemed fit for discharge: absence of nausea or emesis; tolerance to feeding, resumption of bowel function; effective pain management; autonomy in movement and personal hygiene care; absence of clinical or laboratory evidence of postoperative complications.

A surgical examination within 3–5 days and a follow-up dietary examination within 10 days of discharge must be provided. A telephone number must be provided to the patient that can be contacted 24 h a day to report any clinical problems.

## Outcomes

### Primary objective

The main objective is to evaluate the effect of using the ERAS approach on the average length of hospital stay.

### Secondary objectives

The secondary objectives include the assessment of applicability of the ERAS protocol and the consequent effects in terms of postoperative morbidity, length of stay in intensive care unit, reintervention and readmission rate, restoration of bowel function, mortality rate and quality of postoperative recovery.

Table 2 shows primary and secondary endpoints.

**Table 2:** Primary and secondary end-points.

Primary end-point – Mean length of stay calculated from the day of surgery to the day of discharge, with outliers (duration >95th percentile of the distribution) excluded.
Secondary end-points – Adherence to selected ERAS items, assessed as the proportion of patients managed according to the recommendations of the ERAS protocol – Incidence of postoperative complications according to Clavien-Dindo classification within 30 days after surgery – Mean length of ICU stay (number of nights) – Incidence of reinterventions within 30 days after surgery – Incidence of hospital or emergency department readmissions within 30 days after surgery – Time to recovery of bowel function, calculated from the day of surgery – Incidence of death from any cause within 30 days after surgery – Quality score of postoperative recovery as measured by the QoR-15 questionnaire at approximately 48 h after surgery.

## Sampzle size

Assuming a standard deviation of LOS, excluding outliers, of 4.5 days, which is assumed to be equivalent across the two periods defined by the application of the protocol (pre and post), the estimated sample of approximately 300 patients provides more than 80 % power to show a mean reduction in the LOS of 2 days, from 10 to 8 days, with a one-sided alpha error of 5 %. This calculation incorporates the clustering of observations within the same center, assuming an intra-cluster correlation coefficient of less than 7 % (rho=0.07).

## Statistical analysis

Outcomes of continuous nature will be described using mean value and standard deviation, or median and interquartile range, as appropriate. The occurrence of outcomes of dichotomous nature will be described using absolute frequencies and percentages. To account for the clustering of patients within the same center, comparisons of outcomes between the two periods of ERAS protocol implementation will be conducted using generalized linear mixed-effects models. The functional link and the distribution family will be defined based on the distribution of the outcome variable of interest. Comparisons will be adjusted in the models for the peritoneal cancer index (PCI), the surgical complexity (with or without HIPEC), and for any patient potentially unbalanced prognostic factors in the distribution of the two periods.

The coefficient for the effect of the protocol on the mean LOS (primary endpoint) will be presented together with a one-sided 95 % confidence interval (CI), in accordance with the sample size assessment based on the one-tailed test. For coefficients pertaining to secondary endpoints, 95 % confidence intervals (CIs) will be presented on both sides. Exploratory subgroup analyses will be conducted according to peritoneal cancer index (PCI) and surgical complexity (CRS ± HIPEC).

Within the case report form, required fields with mandatory completion have been identified to investigate the application of various pre-, intra-, and post-operative ERAS items. Adherence to these items will be quantified as a percentage, and its impact on key outcomes (length of hospital stay, complications, readmissions, and bowel motility recovery) will be evaluated through a sensitivity analysis using mixed effects models, with adjustments for adherence level.

Additional sensitivity analyses will be performed by imputing missing data using multiple imputation techniques.

## Ethics and data collection

The study will be conducted in accordance with the ethical principles set out in the Declaration of Helsinki and the standards of Good Clinical Practice, ensuring compliance with regulatory requirements for participant data protection. Prior to enrollment, participants will be provided with comprehensive information about the study, both verbally and in the form of a detailed written consent document. Ethical approval for the study was granted by the Ethical Committee “CET Interaziendale Città della Salute e della Scienza di Torino” on 20/11/2024.

The Case Report Form (CRF) is accessible within a dedicated section of the EPICLIN electronic platform (https://new.epiclin.it/it/eras_carcinosi/). EPICLIN, developed and managed by the Clinical Epidemiology Unit, AOU Città della Salute e della Scienza di Torino complies with all security requirements outlined in the General Data Protection Regulation (EU Regulation 2016/679).

For each participant, an individual identifier (ID) will be generated at the respective recruitment center. A first database will store the association between the participant’s personal data and their ID, ensuring the separation and confidentiality of sensitive information. A second database, hosted on the EPICLIN platform, will contain the ID and the clinical data collected for the purposes of the study, without including any personal information about the participant.

The recruitment officer at each center will be responsible for entering the clinical data into the second database. The system utilized is a validated and secure database, fully compliant with current data protection regulations.

## Discussion

The implementation of the ERAS (Enhanced Recovery After Surgery) protocol in the context of cytoreductive surgery (CRS) and hyperthermic intraperitoneal chemotherapy (HIPEC) represents a significant advancement in perioperative care for patients with peritoneal surface malignancies. The EPICH study builds upon the growing body of evidence supporting ERAS principles across various surgical specialties and extends it to a highly complex patient population with unique perioperative challenges.

CRS and HIPEC procedures are associated with substantial physiological stress, high postoperative morbidity, and prolonged recovery periods due to their extensive nature and the immunosuppressive effects of intraperitoneal chemotherapy [21], 22]. In this study, the ERAS approach seeks to mitigate these challenges by integrating multimodal strategies such as prehabilitation, optimized nutritional support, enhanced anesthesia protocols, and early postoperative mobilization.

Existing literature has demonstrated that ERAS protocols improve outcomes by reducing postoperative complications and hospital stays while enhancing patient satisfaction [23]. For example, patients undergoing elective colorectal or gynecological surgeries under ERAS protocols have shown a mean reduction in length of stay by approximately 2 days and fewer postoperative complications compared to conventional care​ [2]. The primary endpoint of the EPICH study aligns with these findings, aiming to confirm similar benefits in patients with peritoneal malignancies.

Despite its potential benefits, the adoption of ERAS in CRS ± HIPEC has faced barriers, including the complexity of the protocol, interdepartmental coordination, and the need for high adherence to multifaceted guidelines. Surveys conducted as part of the EPICH study revealed these challenges, particularly related to the large number of protocol items and the variability in institutional resources [20]. Addressing these issues, the study employed a consensus-based selection of ERAS elements to ensure practicality and efficacy in this specific surgical context.

The EPICH study design, comparing standard perioperative care with ERAS-based management, offers a pragmatic approach to evaluating the protocol’s impact. By focusing on high-consensus items such as prehabilitation, multimodal analgesia, GDFT, early mobilization and feeding, if possible, the study aims to address logistical constraints while preserving core ERAS principles.

Preliminary data from ERAS applications in CRS ± HIPEC suggest promising results. Studies have shown improved bowel function recovery, fewer ICU admissions, and reduced hospital costs without compromising patient safety [19]. Additionally, the integration of preoperative measures such as anemia correction and nutritional optimization aligns with broader evidence linking these interventions to better surgical outcomes.

By incorporating these strategies, the EPICH study is poised to provide robust data supporting the adaptation of ERAS protocols to the complex CRS ± HIPEC setting. If successful, the findings could promote wider adoption and ultimately improve the quality of care for patients with peritoneal metastasis.

While the study design is robust, certain limitations must be acknowledged. The reliance on a multicenter approach introduces potential variability in protocol adherence, which could impact outcomes. Future research should explore the long-term effects of ERAS on patient-reported outcomes, cost-effectiveness, and the potential for further streamlining protocol components.
